# Phenotypic and Genomic Comparison of the Two Most Common ExoU-Positive Pseudomonas aeruginosa Clones, PA14 and ST235

**DOI:** 10.1128/mSystems.01007-20

**Published:** 2020-12-08

**Authors:** Sebastian Fischer, Sarah Dethlefsen, Jens Klockgether, Burkhard Tümmler

**Affiliations:** a Clinical Research Group Molecular Pathology of Cystic Fibrosis and Pseudomonas Genomics, Clinic for Pediatric Pneumology, Allergology and Neonatology, Hannover Medical School, Hannover, Germany; UCSF

**Keywords:** *Pseudomonas aeruginosa*, genomics, high-risk clone, molecular epidemiology, phenotypic variation, population genetics, transcriptomics

## Abstract

Genotyping of 2,882 Pseudomonas aeruginosa isolates that had been collected during the last 40 years identified the ExoU-positive lineages PA14 (ST253) and ST235 as the second and third most frequent clones in the P. aeruginosa population. Both clones were approximately 2-fold more frequently detected in animate habitats than in soil or aquatic habitats. While ST253 clone isolates were causing mainly acute and chronic infections in humans, ST235 isolates had been preferentially collected from hospitalized patients with severe acute infections, particularly, keratitis, urinary tract infections, burn wounds, and ventilator-associated pneumonia. The two major *exoU* clones differed substantially in the composition and flexibility of the accessory genome and by more than 8,000 amino acid sequences. Pronounced sequence variation between orthologs was noted in genes encoding elements of secretion systems and secreted effector molecules, including the type III secretion system, indicating the modes of action of the different clones. When comparing representatives of the two clones in batch culture, the PA14 strain orchestrated the quorum sensing circuitry for the expression of pathogenic traits and stopped growing in batch culture when it entered the stationary phase, but the quorum sensing-deficient ST235 strain expressed high type III secretion activity and continued to grow and to divide. In summary, unrestricted growth, high constitutive type III secretion activity, and facilitated uptake of foreign DNA could be major features that have made ST235 a global high-risk clone associated with poor outcomes of acute nosocomial infections.

**IMPORTANCE** The ubiquitous and metabolically versatile environmental bacterium Pseudomonas aeruginosa can cause infections in a wide variety of hosts, including insects, plants, animals, and humans. P. aeruginosa is one of the ESKAPE (Enterococcus faecium, Staphylococcus aureus, Klebsiella pneumoniae, Acinetobacter baumannii, Pseudomonas aeruginosa, and *Enterobacter* species) pathogens that are the major cause of nosocomial infections in the United States and are a threat all over the world because of their capacity to become increasingly resistant to all available antibiotics. Most experimental work on P. aeruginosa has been performed with reference strains PAO1 and PA14, providing deep insight into key metabolic and regulatory pathways thought to be applicable to all P. aeruginosa strains. However, this comparative study on the two most common *exoU*-positive clones taught us that there are major lineages in the population such as the global high-risk clone ST235 that exhibit uncommon traits of lifestyle, genome mobility, and pathogenicity distinct from those in our knowledge gained from the studies with the reference strains.

## Abstract (precis)

The ubiquitous and metabolically versatile environmental bacterium Pseudomonas aeruginosa can cause infections in a wide variety of hosts, including insects, plants, animals, and humans. P. aeruginosa is one of the ESKAPE (Enterococcus faecium, Staphylococcus aureus, Klebsiella pneumoniae, Acinetobacter baumannii, Pseudomonas aeruginosa, and *Enterobacter* species) pathogens that are the major cause of nosocomial infections in the United States and are a threat all over the world because of their capacity to become increasingly resistant to all available antibiotics. Most experimental work on P. aeruginosa has been performed with reference strains PAO1 and PA14, providing deep insight into key metabolic and regulatory pathways thought to be applicable to all P. aeruginosa strains.

## INTRODUCTION

Pseudomonas aeruginosa is a ubiquitous metabolically versatile gamma-proteobacterium that can thrive in low densities in soil and aquatic habitats and can colonize the surface of animate hosts ranging from worms and flies to plants and mammals (1). Being an opportunistic pathogen, P. aeruginosa causes a wide range of syndromes in humans that can vary from local to systemic, subacute to chronic, and superficial and self-limiting to life-threatening (2, 3). P. aeruginosa is one of the six ESKAPE (Enterococcus faecium, Staphylococcus aureus, Klebsiella pneumoniae, Acinetobacter baumannii, Pseudomonas aeruginosa, and *Enterobacter* species) pathogens that are the major cause of nosocomial infections in the United States and are a threat all over the world because of their capacity to become increasingly resistant to available antibiotics (4–6). As a result, burn wound infections, pneumonia, and sepsis are burdened with high morbidity and lethality (7). Besides these acute infections, P. aeruginosa has succeeded in causing chronic airway infections in predisposed hosts, i.e., patients with cystic fibrosis (CF) (8), bronchiectasis (9), or chronic obstructive pulmonary disease (COPD) (10).

Based on sequence diversity of the core genome as the distinctive feature, whole-genome sequencing projects have demonstrated that the current P. aeruginosa population consists of two major groups, 1 and 2, and three small groups of distant outliers (11–14). The groups differ in their repertoire of the four effector proteins (ExoS, ExoT, ExoU, and ExoY) that are secreted by the type III secretion system (T3SS), the major virulence determinant of P. aeruginosa for mammalian hosts (1, 15). The presence of the *exoS* gene is characteristic of P. aeruginosa isolates of group 1, whereas the presence of the *exoU* gene is characteristic of P. aeruginosa isolates of group 2 (12, 13). Of the effector proteins delivered by the T3SS, ExoU is the most toxic (15). ExoU possesses potent phospholipase activity, which causes rapid cell lysis and necroptosis of mammalian cells (16). In comparison to strains expressing ExoS, ExoU-expressing P. aeruginosa strains have been associated with more severe outcomes in human infections (16).

In this report, we compare epidemiological, genomic, and phenotypic features of the two most common *exoU*-positive clones in the P. aeruginosa population (17) that make up approximately 40% of group 2 isolates. Clone D421 (ST253), having strain PA14 as a thoroughly studied representative (18–20), expressed the typical traits that we expect for P. aeruginosa. Conversely, clone F46D carried an uncommon genetic cargo and mutation spectrum in its genome. The conveyed gain-of-function and loss-of-function characteristics may explain why F46D, better known in the literature as ST235 (21, 22), has become the major global high-risk clone in the current P. aeruginosa population.

## RESULTS

### Population biology of P. aeruginosa.

During the last 40 years, we have collected more than 6,000 P. aeruginosa isolates primarily from human infections and the inanimate aquatic environment. The majority of strains were from sites in Germany, but substantial portions were also received from other European countries, Asia, Australia, and North America (Table 1). By July 2020, 2,882 isolates had been genotyped by a custom-made microarray that represents the conserved core genome with 16 single nucleotide polymorphisms (SNPs) and the variable accessory genome with 42 marker genes, including *exoS* and *exoU* (23) (see Data Set S1 in the supplemental material). Consistent with whole-genome sequencing studies (11, 12), we observed two major clades for strains positive for *exoS* (78.0%) or *exoU* (19.2%) and a minor clade (2.8%) that is negative for these type III secretion virulence effectors. The two most common *exoU*-positive clones, D421 and F46D (the latter previously assigned according to the hybridization signal pattern as F469 [17, 23]), were the second and third most frequent clones in our collection making up 40.6% of *exoU*-positive isolates. Both clones were approximately 2-fold more frequently detected in animate habitats than in soil or aquatic habitats (Table 2).

**TABLE 1:** Frequency distribution of the geographic origin of isolates of the Hannover P. aeruginosa strain collection from independent habitats

Habitat	Frequency (%)		
	Germany	Other European countries	Non-European countries
Total (all habitats)	64	22	14
Inanimate environment	74	6	20
Acute infection	30	63	7
Chronic airway infection	74	12	14

**TABLE 2:** Hannover strain collection: epidemiology of P. aeruginosa clones ST253 (D421) and ST235 (F46D)^*a*^

Habitat	ST253		ST235	
	Detection frequency (%)	Rank	Detection frequency (%)	Rank
Inanimate environment	2.1	9	0.7	32
Acute infection	3.9	4	11.1	1
Chronic airway infection in:				
COPD patients	8.1	2	3.2	3
CF patients	4.3	2	1.3	16
Total (all habitats)	3.8	2	3.6	3
Acute human infections with P. aeruginosa				
Intensive care units				
Acute pneumonia	4.0	6	17.8	1
Burn wounds	6.7	5	40.0	1
Ulcerative keratitis	6.3	4	12.5	1
Urinary tract infection	7.7	3	10.8	1
Other acute infection^*b*^			3.4	

a The collection of ST235 and ST253 isolates does not contain any MDR or XDR strain. Shown are detection frequencies and rank within the habitat.

b Including wound isolates not treated at an intensive care unit.

10.1128/mSystems.01007-20.1 DATA SET S1 P. aeruginosa genotypes of all isolates. The table lists the habitat, geographic origin, date of isolation, hexadecimal clone type, and 58-marker genotype of 2,882 P. aeruginosa strains. If applicable, strain names and information about habitat and geographical and temporal origins are also given. Download Data Set S1, XLS file, 1.8 MB. Copyright © 2020 Fischer et al. 2020 Fischer et al. This content is distributed under the terms of the Creative Commons Attribution 4.0 International license.

### Matching microarray and MLST genotypes.

The oligonucleotide microarray types P. aeruginosa strains according to the conserved core genome determining the clonal lineage and according to the flexible accessory genome (23). Alternatively, P. aeruginosa strains are frequently typed by multilocus sequence typing (MLST) in seven housekeeping genes (24). To determine the correspondence between microarray genotype and MLST sequence type, we sequenced the genomes of 13 clone D421 and 13 F46D strains and determined the hexadecimal array code from sequenced P. aeruginosa strains deposited in the PATRIC (25) and *Pseudomonas* (26) databases. Clone D421 represented by reference strain PA14 (18, 19, 27) and the early CF isolate RN3 (28) correspond to sequence type ST253 in the MLST database. On the other hand, when we extracted the array genotype from 11 sequenced ST253 strains, eight strains belonged to clone D421 and the other three strains to the closely related clone D021. Twelve of the 13 F46D strains, including the COPD isolate 60P57PA (29), turned out to be sequence type ST235 that is currently classified as the most prevalent global high-risk clone associated with multidrug resistance to carbapenems, aminoglycosides, and fluoroquinolones (21, 22). Vice versa, 34 sequenced ST235 strains collected worldwide from hospitals in 16 countries were consistently genotyped *in silico* to be F46D. One sequenced F46D isolate from our strain collection was assigned to ST205, and another eight F42D isolates that differ from F46D in one of the 16 array SNPs were assigned to, in total, six sequence types (ST313, ST319, ST377, ST815, ST823, and ST830). In summary, the majority of D421 and F46D isolates belong to ST253 and ST235, respectively.

### Infection epidemiology of P. aeruginosa ST235 and ST253.

In our strain collection, which to our knowledge, is currently the largest single-site collection of P. aeruginosa isolates from the last 40 years, ST253 strains had been isolated at similar rates from acute and chronic human infections, whereas ST235 was the most frequent clone in acute infections (Table 2; Data Set S1). ST253 was the second and third most frequent clone among isolates from CF and COPD patients, respectively, who all were chronically harboring ST253 strains in their airways for years to decades. In contrast, the ST235 strains occurred as singular or short-term colonizing isolates in CF and COPD patients who all spontaneously cleared P. aeruginosa ST235 from their airways without antimicrobial intervention. These clinical data suggest that members of the ST253 clone are proficient chronic colonizers of CF and COPD lungs, whereas members of the ST235 clone exhibit low pathogenicity in these individuals who are predisposed to chronic lung infection with P. aeruginosa. On the other hand, ST235 was a high-risk clone in individuals who became infected with P. aeruginosa during an acute critical illness (Table 2). ST235 was the most abundant clone in severe eye and urinary tract infections and in critically ill patients accommodated in intensive care units (ICUs) suffering from ventilator-associated pneumonia or infections of extensive burn wounds.

### Comparative genomics of the ST253 and ST235 clones.

The first complete genome sequences of representatives of the ST253 and ST235 clones were reported for the burn wound isolate PA14 (27) and the multidrug-resistant (MDR) airway isolate NCGM2.S1 (30), respectively. To allow both intra- and interclonal genome comparisons, we included completely sequenced genomes from the databases and the genomes of 26 isolates from our strain collection as further representatives of clones ST235 and ST253.

First, we compared the shared core genome among the strains. Within each clone, the nucleotide sequence diversity compared to the reference strains PA14 and NCGM2.S1 varied between 75 and 499 nucleotides (median, 136 nucleotides [nt]) for ST253 (D421) and between 138 and 284 nucleotides (median, 127 nt) for ST235. Thus, for both clones, the within-clone core genome sequence diversity is approximately 2,000-fold lower than the average interclonal sequence diversity in P. aeruginosa of approximately 0.5% (13). The ST253 (D021) genomes, however, showed a sequence diversity of 0.03% with that of PA14, confirming their above-mentioned assignment as being closely related to, but not matching with, ST253 (D421).

**FIG 1:**
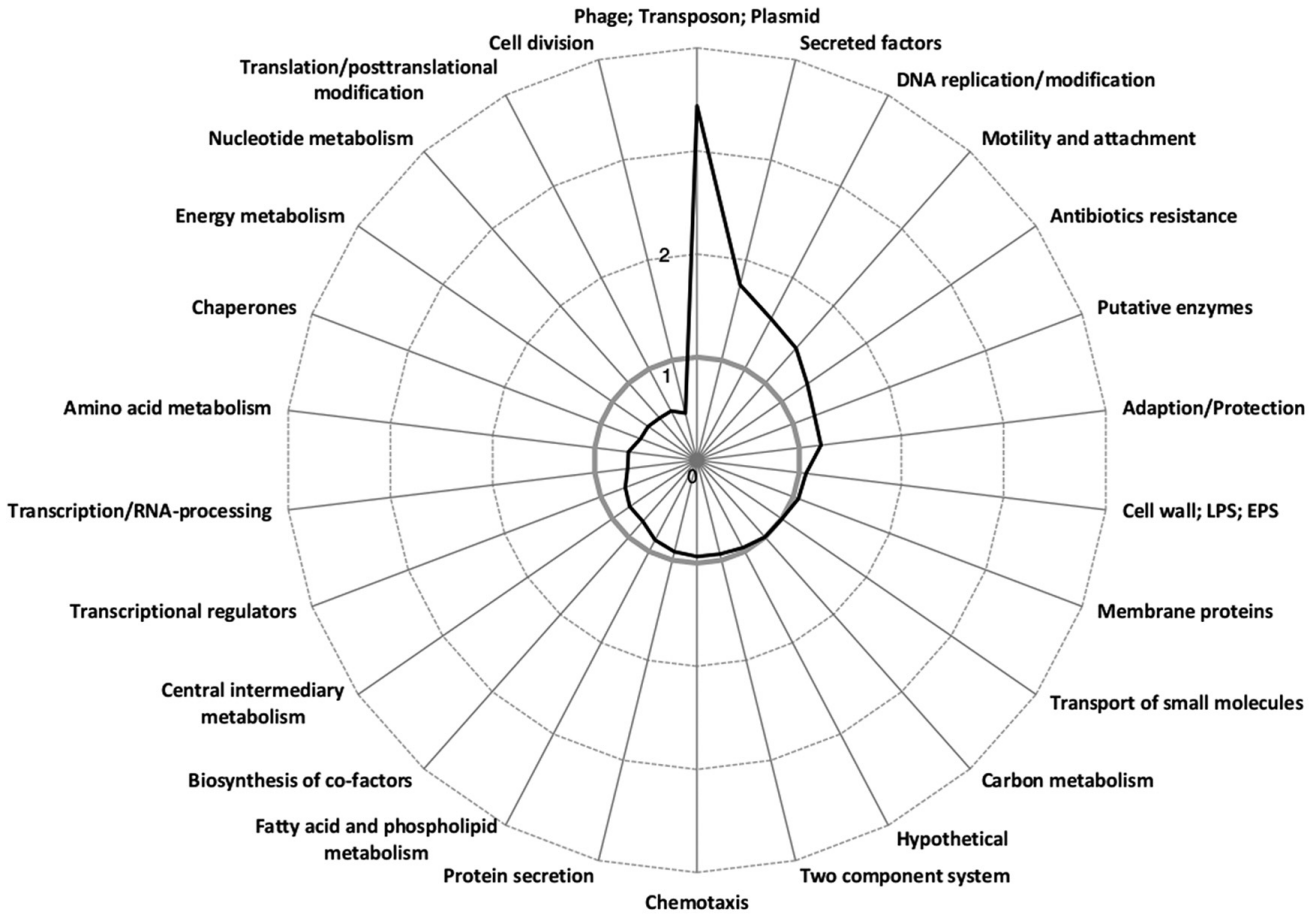
Amino acid sequence variation between the genomes of P. aeruginosa strains PA14 (ST253) and 60P57PA (ST235) sorted by functional category. The reads of strain 60P57PA (ST235) were mapped to the PA14 reference strain (ST235), and 8,150 amino acid differences were detected and functional categories were assigned according to PA14 annotation. As a comparison, the frequency was compared to the distribution in the PAO1 reference genome by dividing the detected functional classes by the number of occurrences of the respective class within the PAO1 reference genome. The gray line symbolizes a theoretical even distribution between the number of amino acid exchanges in ST235 compared to that in ST253 and the occurrence of functional classes in PAO1. EPS, exopolysaccharide.

Amino acid sequences were different at more than 8,000 positions between ST235 and ST253 strains (see Data Set S2). The largest amino acid sequence diversity was recognized among the genes of the functional categories that encode the elements of DNA replication, antibiotics resistance, secreted factors, and motility and genome mobility, such as phages and transposons, respectively (Fig. 1; Data Set S2). On the other hand, coding sequence variants were underrepresented in the functional categories of cell division and translation. Reciprocal read mapping identified the 141 orthologous genes with the largest sequence variation between ST235 and ST253 (see Data Set S3). Sequence variation was pronounced in yet-uncharacterized hypotheticals, mercuric resistance and conjugal transfer operons, and genes of the lipopolysaccharide (LPS), pyoverdine, and flagellum and pilus biosynthesis clusters (Data Set S3). We moreover searched for the genes with the most drastic differences in the coding sequence between ST235 strains and the PA14 reference. Of all 34 genes listed in Data Set S4 that encode an element of a secretion system or a secreted effector molecule, drastic amino acid variants indicated by a Dayhoff score of <4 were noted in 14 genes, including those encoding the major virulence effector exotoxin A and the major virulence effector of the type III secretion system, the patatin-like phospholipase exotoxin U. Of the other T3SS genes, the ST235 clone has an amino acid variant of exoenzyme S and a variant of exoenzyme T harboring other amino acids at six positions and an extended C-terminal sequence caused by the conversion of the termination into a glutamine codon. The T3SS adenylate cyclase effector gene *exoY* is either truncated or absent in ST235 strains. C-terminal extensions of the gene product are also caused by stop codon mutations in *hbcR*, encoding a transcriptional regulator, and *cat*, the chloramphenicol transferase gene. Conversely, a premature stop codon was observed in an ECF-σ^70^ sigma factor (PA1363). In summary, the core genomes of ST235 and ST253 differ in the coding sequence of genes encoding elements of secretion, motility, and virulence.

10.1128/mSystems.01007-20.2 DATA SET S2 List of 8,150 clone-specific amino acid exchanges in the coding sequence of strain NCGM2.S1 (ST235) compared to that for the PA14 (ST253) reference strain. The table lists the nucleotide substitution, its genome position in the PA14 genome, the resulting amino acid substitution, and the affected locus. The table, moreover, flags the hotspots of clone-specific amino acid sequence variants between ST235 and ST253 with five or more nonsynonymous substitutions within a gene. Download Data Set S2, XLSX file, 0.4 MB. Copyright © 2020 Fischer et al. 2020 Fischer et al. This content is distributed under the terms of the Creative Commons Attribution 4.0 International license.

10.1128/mSystems.01007-20.3 DATA SET S3 Orthologous gene loci exhibiting the overall largest sequence divergence between the reference strains PA14 (ST253) and NCGM2.S1 (ST235). Download Data Set S3, XLSX file, 0.1 MB. Copyright © 2020 Fischer et al. 2020 Fischer et al. This content is distributed under the terms of the Creative Commons Attribution 4.0 International license.

10.1128/mSystems.01007-20.4 DATA SET S4 Orthologous genes in ST235 that, in comparison to reference strain PA14 of ST253, exhibit phenotypically relevant nucleotide substitutions, i.e., drastic amino acid changes (Dayhoff score < 4) or frameshift mutations, or upstream or downstream shifts of the termination codon. Within-clone diversity of these drastic mutations is shown for 13 completely sequenced ST235 strains. Green background color indicates a match with the consensus information provided in the left columns. Red background color highlights divergent features of the individual strain. Download Data Set S4, XLS file, 0.1 MB. Copyright © 2020 Fischer et al. 2020 Fischer et al. This content is distributed under the terms of the Creative Commons Attribution 4.0 International license.

**FIG 2:**
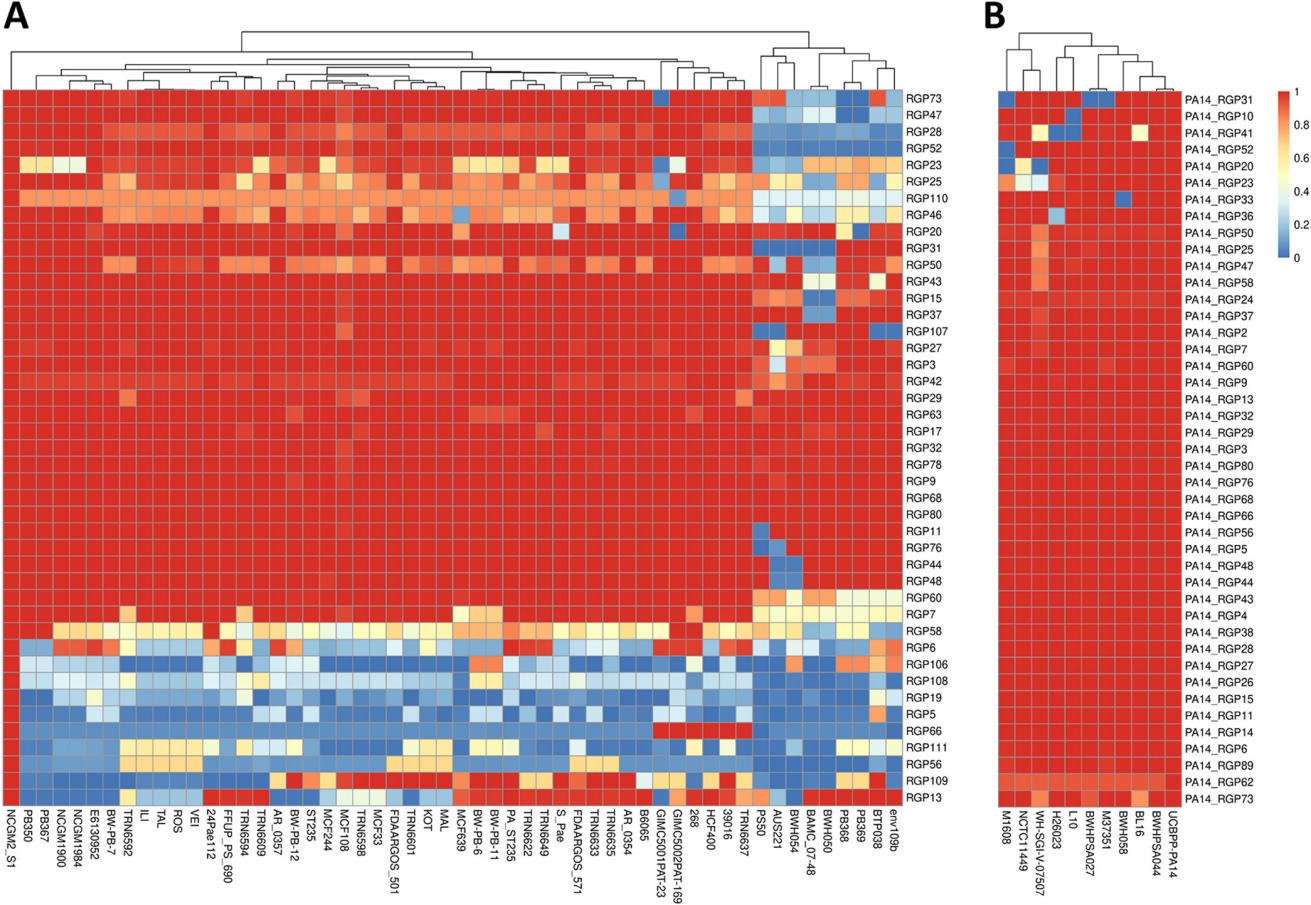
Compositions of the accessory genomes of P. aeruginosa ST235 and ST253 strains. (A) The heat map shows the compositions of the accessory genomes in terms of RGPs of ST235 (F46D) isolates as well as of unrelated sequence types with a similar hexadecimal code. The dendrogram indicates that the ST235 isolates (left) are distinct from other sequence types (right outermost group of nine strains: ST205, AUS221; ST313, BAMC_07-48 and BWH050; ST319, PB368 and PB369; ST377, PS50; ST815, env109B; ST823, BTP038; ST830, BWH054) by their composition of accessory elements. The heat map depicts seven yet undescribed RGPs which were designated RGP106 to RGP112. Genome coordinates are given in Data Set S8 in the supplemental material. (B) The heat map shows the compositions of the accessory genomes in terms of RGPs in ST253 isolates whose genomes are represented in databases by less than 100 contigs. The ST253 variants of hexadecimal code D421 and D021 differ from each other by the presence and absence of RGP31. The barcode indicates the sequence similarity with the respective RGP of reference strains NCGM2.S1 (A) and PA14 (B).

DNA insertions in “regions of genome plasticity” (RGPs) define the accessory genome (31). The heat maps in Fig. 2 provide an overview about the composition of the accessory genomes in ST235 and ST253. Figure 2A depicts the accessory genome of 52 F46D strains, the left 43 strains belonging to ST235 and the rightmost nine strains to other sequence types. The latter nine strains are clearly separated in their accessory genome from that of ST235. The ST235 strains share approximately 20 RGPs but show strain-to-strain variation in the compositions of a further six RGPs. Reference NCGM2.S1 harbors 12 RGPs that are present in only a few other strains, classifying them as rather strain specific. Conversely, clone ST253 is characterized by a more homogeneous accessory genome (Fig. 2B). Compared to those in the PA14 reference, only few RGPs are absent or have a different composition in the other ST253 strains.

Next, we compared the constituents of the accessory genomes of the two clones. The examined ST235 60P57PA and ST253 PA14 strains share identical insertions in 14 RGPs (mostly smaller than 10 kbp) and display partially conserved DNA in some more, but the majority of the RGP insertions differ between the two clones (see Data Set S5), suggesting a different makeup of the accessory genomes. Of the 29 (ST253) or 25 (ST235) individual accessory elements, the majority were larger than 10 kbp in size and included all replacement islands: the two clones display different types of flagellum glycosylation genes, *pilA* genes, serotype-defining LPS biosynthesis gene clusters, and pyoverdine biosynthesis genes. The *exoU* gene is found in both clones in the same genomic region (RGP7) but embedded in different types of ExoU islands (32). CRISPR/Cas systems are present in ST253 strains but absent in ST235 strains. The 22 previously reported ST235-specific genes which cluster into three blocks (22) were also 100% conserved in all 13 drug-sensitive ST235 isolates of our collection. Block 2 carries nine genes encoding proteins implicated in DNA processing, including *dprA* and *recQ*, which belong to the transformation machinery of naturally transformable bacterial species (22).

10.1128/mSystems.01007-20.5 DATA SET S5 Accessory DNA of representatives of clones ST253 (strain PA14) and ST235 (strain 60P57PA) in P. aeruginosa regions of genome plasticity (RGPs). RGPs are genomic regions in which accessory DNA was detected in one or several P. aeruginosa genomes (31). One hundred one RPGs have been characterized by the conserved (core genome) flanking open reading frames (ORFs) and, if identified, by the exact insertion sequences (attachment sites) such as tRNA genes. The 101 characterized RGPs were given numbers between 1 and 111 (please note that some numbers were not used in the initial RGP descriptions). Download Data Set S5, XLSX file, 0.1 MB. Copyright © 2020 Fischer et al. 2020 Fischer et al. This content is distributed under the terms of the Creative Commons Attribution 4.0 International license.

### Phenotypes.

**FIG 3:**
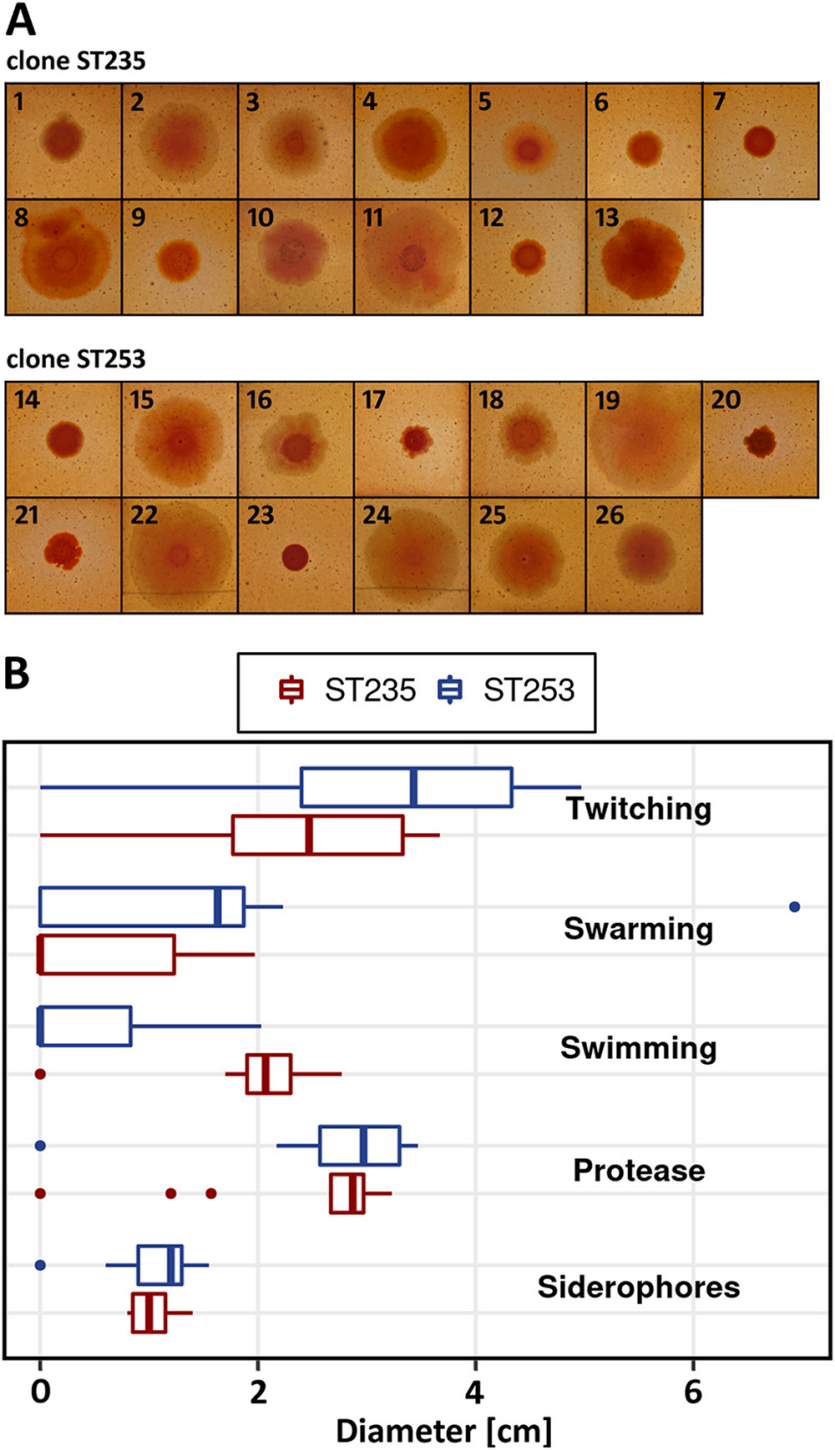
Phenotypic comparisons of ST235 and ST253 isolates. (A) Colony morphology after growth on Congo red agar plates. (B) Boxplot summarizing motility and secretion assay results for the two clonal lineages. Displayed diameter values represent the mean size of motility zones, or halos formed by protease and siderophore secretion, on the corresponding agar plates. Motility and secretion assays were performed in triplicates for each tested isolate. For each clone, 13 isolates were tested. Clone ST235: 1, 60P57PA; 2, W15Dec14; 3, BW-PB-6; 4, BW-PB-7; 5, BW-PB-11; 6, BW-PB-12; 7, AI-BN-7; 8, B6065; 9, MCF33 (a); 10, MCF108 (a); 11, MCF244 (a); 12, MCF639 (a); 13, HCF400 (a). Clone ST253: 14, PA14; 15, 110E8; 16, K4; 17, K7; 18, HCF202 (a); 19, HCF209; 20, MCF228 (a); 21, Zw61 (a); 22, Zw86_2; 23, MCF37; 24, SS8; 25, 012SA1; 26, HCF212. Isolates marked with (a) displayed autolysis upon growth on blood agar plates. Origin and isolation information for these isolates is included in Data Set S1.

The colony phenotypes of panels of 13 ST235 and 13 ST253 strains were assessed in plate-based assays (Fig. 3). The strains were diverse in colony morphology and motility but similarly proficient in the secretion of protease and siderophores. We noted no association between clone and phenotype, the exception being a consistently higher capability of the ST235 strains for swimming.

**FIG 4:**
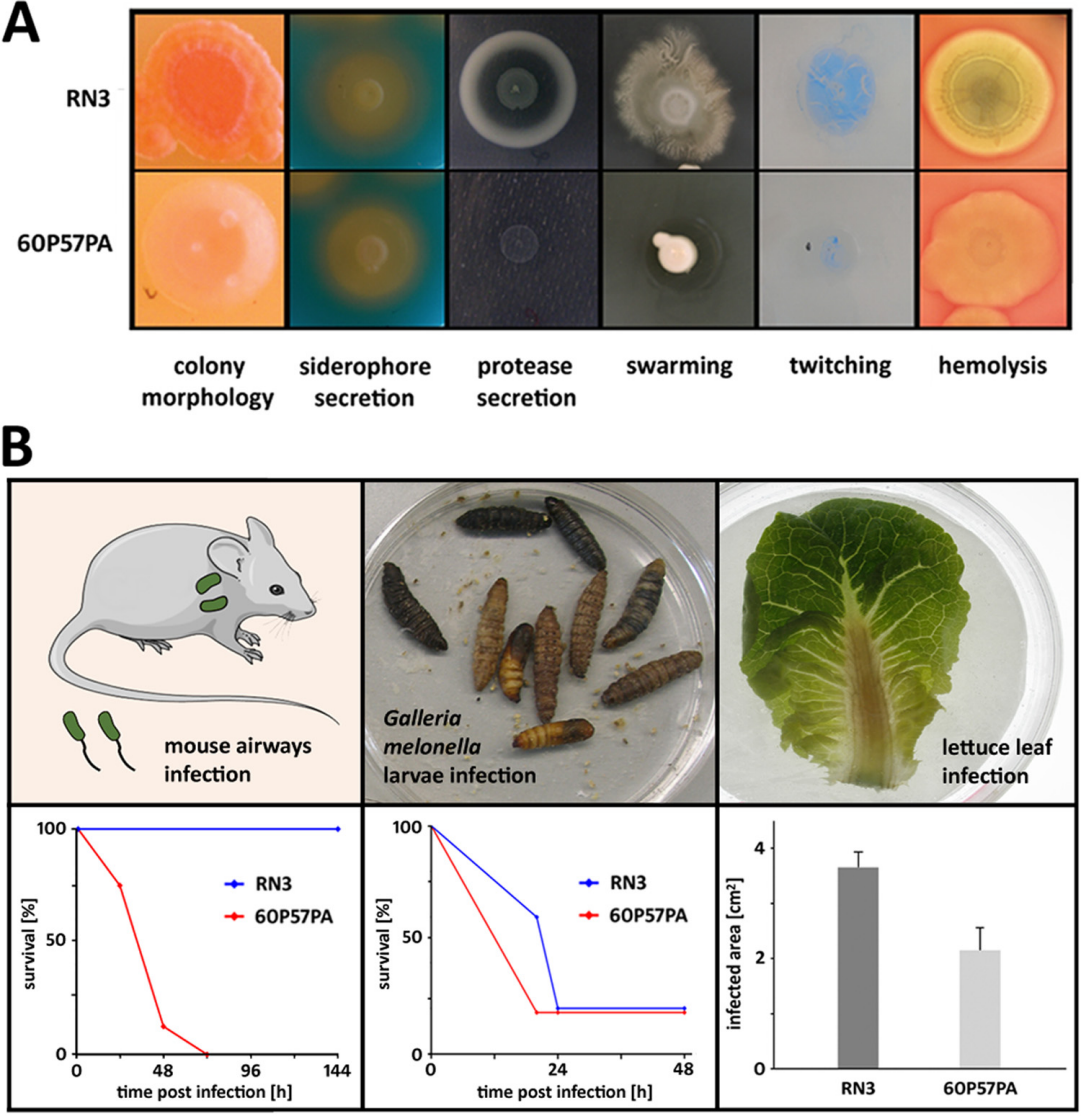
Phenotype and virulence comparisons of ST253 isolate RN3 and ST235 isolate 60P57PA that had been selected for growth characteristics and transcriptome comparison. (A) Plate assays for motility and secretion phenotypes. (B) Outcomes of three different virulence models (mammalian, nonmammalian, and plant infection). The results were taken from a previous study on virulence and genomic comparisons of representatives from 20 P. aeruginosa clonal lineages (13).

We previously sequenced ST253 strain RN3 and ST235 strain 60P57PA together with representatives of 18 other common P. aeruginosa clones and tested their virulence in mammalian, invertebrate, and plant infection models (13). The pathogenicity of RN3 was intermediate within the strain panel for all three infections, whereas the ST235 isolate 60P57PA was nonvirulent in the lettuce leaf infection, slightly pathogenic in the wax moth infection, and the most virulent strain in the acute murine airway infection model (Fig. 4B) (13). In addition to this previous work, we now examined the phenotype of the two strains on agar plates. RN3 showed the typical characteristics of P. aeruginosa, i.e., it was motile, induced hemolysis, and secreted pigments, siderophores, and proteases. Conversely, strain 60P57PA was colorless and deficient in motility and protease secretion (Fig. 4A).

### Growth behavior during fermentation *in vitro*.

**FIG 5:**
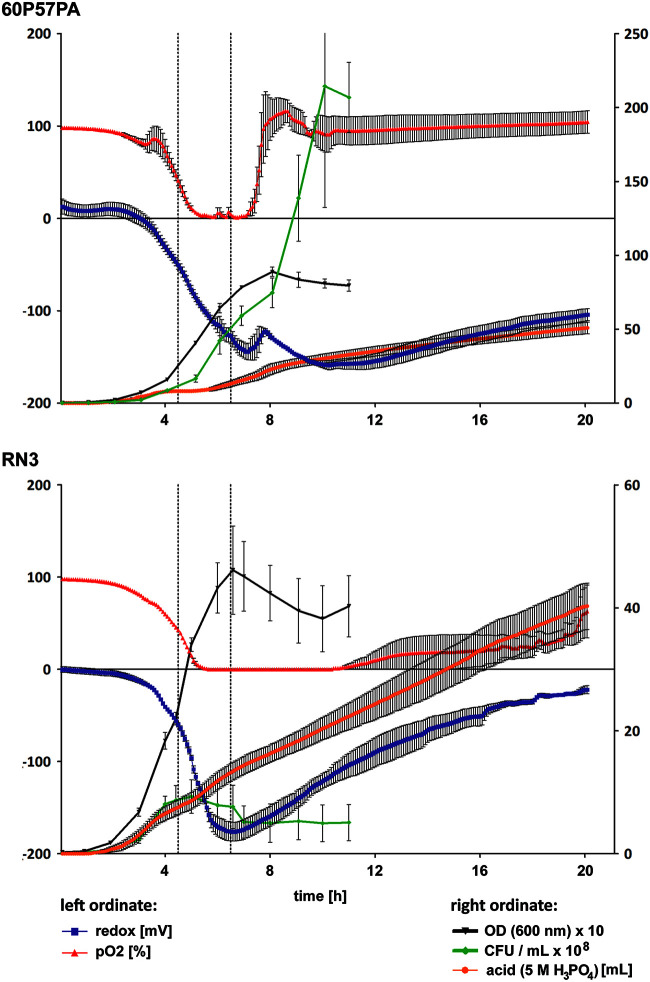
Growth of clone ST235 strain 60P57PA (top) and of clone ST253 strain RN3 (bottom) in batch culture. Bacteria were grown in 1.5 liters tryptone soy broth at 37°C and pH 7.0 with constant agitation (400 rpm) and supply of 0.3 liters/min compressed air. Oxygen saturation (red), redox potential (purple) (left scale), and the supply of H_3_PO_4_ to maintain pH 7 (right scale) were continuously monitored. Every 60 min, 1 ml was removed from the culture for the determination of OD_600_ (black) and CFU (green) (right scale). Fermentations were executed twice. The two dotted lines indicate the time points when samples were taken for RNA-seq analysis of the bacterial transcriptome.

Next, we compared the growth of these strains under standardized conditions in a fermenter. The RN3 and 60P57PA bacteria were cultured in tryptone soy broth at 37°C and pH 7.0 under constant agitation and with a continuous supply of compressed air. Strain RN3 multiplied within the first 5 h to a maximal cell density of 10^9^ CFU/ml (Fig. 5). By that time point, oxygen saturation had dropped from initially 100% to zero. Thereafter, CFU decreased by approximately 3-fold within the next 2 h and then remained constant for a further 4 h. Strain 60P57PA initially showed a growth behavior like that of strain RN3, but from the fifth hour of fermentation, it became more and more divergent. Oxygen saturation declined from 100% to zero within the first 5 h, remained at zero for the next 2 h, but thereafter increased to 100% within an hour and remained at that level until the end of the 20-h fermentation (Fig. 5). Strain 60P57PA apparently had changed its lifestyle, so that the supplied oxygen was not consumed anymore. In parallel, the number of CFU increased continuously, leading to a maximal cell density of 2 × 10^10^ CFU/ml, which is 100-fold higher than that obtained for strain RN3 cultured under the same conditions. The different growth behaviors of the strains also showed up in the divergent time courses of turbidity and CFU. RN3 showed a stronger increase of optical density (OD) than of CFU during late exponential and stationary phases, indicating the release of turbid inanimate material, most likely exopolysaccharides and cell debris. In contrast, the steeper increase of CFU than that of OD during growth of 60P57PA probably reflects bacterial growth in clumpy microcolonies. In summary, the two strains showed highly divergent growth characteristics in batch culture.

### Comparative transcriptomics of growth in fermenter.

The global gene expression profile of RN3 and 60P57PA in the batch cultures was determined by transcriptome sequencing (RNA-seq) during mid-exponential phase and 2 h later during the transition to stationary growth. The time points of sampling are indicated by dotted lines in Fig. 5. When we compared the two time points for strain RN3, 36 and 41 genes were significantly upregulated in mid-exponential and early stationary phases, respectively (see Data Set S6). During exponential growth, RN3 more strongly expressed the genes for ribosomal proteins, transport of amino acids, and catabolism of arginine, consistent with the provision of tryptone soy broth as nutrients. During stationary phase, the RN3 cells then upregulated the ribosome modulation factor and elements of the quorum sensing circuitry but also numerous hypotheticals of unknown function. When we compared the transcriptome of exponential and stationary growth of 60P57PA, approximately 3-fold more significantly differentially regulated genes were identified in 60P57PA than in RN3. During exponential phase, 60P57PA more strongly expressed numerous mRNA transcripts for central elements of transcription, translation, and energy and central intermediary metabolism without any bias toward amino acid metabolism, as was observed for RN3 (Data Set S6). Two hours later, the 60P57PA population had switched to the expression of other functional categories, for example, genes involved in signaling, chemotaxis, and catabolism of aromatic compounds. The majority of genes, however, were hypotheticals of unknown function (Data Set S6).

10.1128/mSystems.01007-20.6 DATA SET S6 RNA-seq analysis of mRNA transcript levels of ST253 strain RN3 and ST235 strain 60P57PA during growth in batch culture. Samples were collected during mid-exponential phase (ME) and during the transition to stationary growth (ES). Time points of sampling are indicated in Fig. 5 by dotted lines. The RNA-seq data sets are available from the European Nucleotide Archive (ENA) hosted by the European Bioinformatics Institute EMBL-EBI (www.ebi.ac.uk; accession no. PRJEB37218). Intraclonal comparison of significantly higher transcript levels between the two time points of sampling (ME versus ES) for strain RN3 and strain 60P57PA. Download Data Set S6, XLSX file, 0.1 MB. Copyright © 2020 Fischer et al. 2020 Fischer et al. This content is distributed under the terms of the Creative Commons Attribution 4.0 International license.

Next, we compared the differential mRNA transcript expression profiles between the two strains (see Data Set S7). In strain RN3, significantly higher transcript levels were observed at both time points for phenazine biosynthesis and the quinolone and quorum sensing networks. Strain 60P57PA more strongly expressed members of the type III secretion network, including ExoU at both time points. Moreover, transcript levels were elevated in early stationary phase for the catabolism of amino acids and aromatic compounds (Data Set S7). In summary, the two strains predominantly differed from each other in their transcriptional profiles of quorum sensing and type III secretion.

10.1128/mSystems.01007-20.7 DATA SET S7 RNA-seq analysis of mRNA transcript levels of ST253 strain RN3 and ST235 strain 60P57PA during growth in batch culture. Samples were collected during mid-exponential phase (ME) and during the transition to stationary growth (ES). Time points of sampling are indicated in Fig. 5 by dotted lines. The RNA-seq data sets are available from the ENA under accession no. (PRJEB37218). Interclonal comparison of significantly higher transcript levels at the same time point of growth phase (ME or ES) between strain RN3 and strain 60P57PA. Download Data Set S7, XLSX file, 0.1 MB. Copyright © 2020 Fischer et al. 2020 Fischer et al. This content is distributed under the terms of the Creative Commons Attribution 4.0 International license.

10.1128/mSystems.01007-20.8 DATA SET S8 Characterization of novel RGPs. Download Data Set S8, XLSX file, 0.1 MB. Copyright © 2020 Fischer et al. 2020 Fischer et al. This content is distributed under the terms of the Creative Commons Attribution 4.0 International license.

## DISCUSSION

The global P. aeruginosa population consists of five groups, of which, 98% of strains belong to the T3SS-positive groups 1 and 2 (12). The T3SS virulence effector ExoS was present in 100% of group 1 and 3% of group 2 isolates, whereas ExoU was exclusively present in 94% of group 2 isolates (12). Clone C (C40A) is the most abundant genotype among the ExoS-positive group 1 clones (17), but for decades, experimental work has mainly been performed with a representative of a rare group 1 clone, i.e., strain PAO1, a burn wound isolate from the 1950s. PAO1 has been and still is the prototype to resolve the physiology, metabolism, and genome organization of P. aeruginosa (33, 34). The group 2 strain UCBPP-PA14, a burn wound isolate from the early 1970s (18, 19), has become the second major reference strain in P. aeruginosa research. Being initially introduced as a strain that exhibits highly virulent properties in both animals and plants due to several common pathogenicity factors (20), the PA14 strain has become a major workhorse to investigate virulence, biofilms, signaling, or quorum sensing (subject of 62% of publications, PubMed, search term Pseudomonas aeruginosa PA14, assessed 6 March 2020). PA14 belongs to the most frequent ExoU-positive clone ST253 characterized by a highly conserved core genome of 0.002% sequence diversity (35). Thus, our current knowledge about P. aeruginosa has been substantially shaped by studies with this ST253 strain, particularly because the valuable resource of an ordered transposon library has been provided to the *Pseudomonas* community (36). This work now introduces the high-risk clone ST235 to be the third most common clone in the P. aeruginosa population. So far, almost all publications have dealt with the infection epidemiology and antimicrobial resistance of the ExoU-positive ST235 clone (subject of 88% of publications, PubMed, search term Pseudomonas aeruginosa ST235, assessed 6 March 2020) (37–49). Antibiotic resistance determinants of MDR and extensively drug-resistant (XDR) ST235 strains have been characterized in detail (40–43, 45–47, 49); otherwise, our current knowledge about the lifestyle, physiology, and metabolism of the pandemic ST235 clone is scarce (50).

We have collected thousands of P. aeruginosa isolates during the last 40 years. An oligonucleotide microarray was developed that types P. aeruginosa strains according to both the conserved core and the flexible accessory genome (23). Whole-genome sequence alignments allowed us to match the microarray genotypes and MLST sequence types of strains. The most common ExoU genotypes D421 and F46D in our collection corresponded in more than 80% of the examined strains to the MLST sequence types 253 and 235, respectively. The in total more than 200 D421 and F46D isolates in our collection originate from 27 different geographic areas in Japan, the United States, and 10 European countries. Hence, our data for these two genotypes should not be strongly biased by geographic origin.

Most ST235 strains were collected between 1998 and 2007. They are susceptible to the common antipseudomonal agents in contrast to the ST235 isolates reported from the last decade that were typically either multidrug or extensively drug resistant (21, 22, 37–49). Thus, our strains may be of interest for the research community to study the unusual features of ST235 in a normal antibiotic-sensitive background. Like the contemporary strains, these early ST235 isolates lack a CRISPR/Cas defense system against phage and carry the clone-specific genes that encode proteins implicated in DNA processing and bacterial transformation (22). The drug-sensitive strains, like their MDR descendants, were already genetically proficient for the uptake and integration of foreign DNA.

Likewise, these drug-sensitive isolates turned out to be highly pathogenic. Most strains were collected from immunocompetent inpatients who were suffering from acute severe or even life-threatening *Pseudomonas* infections of lungs (51), eyes (52), or burn wounds (53). This scenario will require antipseudomonal chemotherapy that inherently bears a higher risk of the development of resistance. High virulence and the propensity to take up DNA, such as antibiotic resistance determinants, make it plausible why ST235 has become a global high-risk clone for severe hospital infections, particularly, at the ICU. In contrast, the ST253 clonal isolates preferentially caused chronic airway infections in patients with bronchiectasis (54), COPD (29), or CF (55, 56). ST253 and ST235 apparently differ in their affinity to human hosts. ST235 attacks the immunocompetent host who is affected by a severe acute trauma, whereas ST253 chronically colonizes the predamaged or predisposed host.

Our comparative genomics and RNA-seq study provides some hints to the etiology of the divergent modes of pathogenicity of the most abundant ExoU-positive clones. Clones ST235 and ST253 differ in the compositions and flexibility of the accessory genome and by more than 8,000 amino acid sequence variants in the core genome. The most extensive sequence variation between orthologs was noted in genes encoding elements of secretion systems and secreted effector molecules, indicating strong diversifying selection in these loci. ST235 has been estimated to be a young clone that emerged in the 1980s (22), and apparently, it has rapidly optimized its repertoire to attack the acutely vulnerable immunocompetent mammalian host. Consistent with this interpretation, exotoxin A and the T3SS effectors ExoT, ExoU, and ExoY belong to the 34 orthologs least conserved between ST235 and ST253. ST235 strains carry an extended version of ExoT and no or truncated ExoY. Only the full-length ExoY is a highly active toxin to exert its cytopathic effects (57), but this activity seems to be dispensable in strains whose virulence is driven by the cytotoxic activity of ExoU. The T3SS is typically activated in the presence of eukaryotic cells. Hence, it was unexpected that T3SS was active in the ST235 strain 60P57PA during fermentation in the absence of any target or competitor. 60P57PA, moreover, showed unusual growth characteristics in the batch culture. If this behavior were typical for all ST235 strains, it could explain the success of this clone in acute severe infections. The investigated ST253 strain stopped growing when it entered stationary phase, but the quorum sensing-deficient ST235 strain switched its lifestyle when nutrients became scarce. The ST235 strain did not consume oxygen, apparently adapted to a microaerophilic lifestyle, and continued to grow and to divide. In summary, unrestricted growth, high T3SS activity, and facilitated uptake of foreign DNA could be the major features that have made ST235 a global high-risk clone associated with poor outcomes of acute nosocomial infections.

### Conclusion.

Strains PAO1 (1, 33, 34) and PA14 (18, 20, 27) provided deep insights into key metabolic and regulatory pathways thought to be applicable to all P. aeruginosa, but this comparative study taught us that within a few years, major lineages can emerge that exhibit traits of lifestyle and pathogenicity uncommon for an ordinary P. aeruginosa. According to genome sequence data, ST235 is a rather young P. aeruginosa clone that emerged in the early 1980s, split into two groups at the turn of the millennium (22), and today is the most frequent clone in acute human infections. The combination of mutation and import of sequences initially generated clone-specific signatures of lifestyle, genome mobility, and virulence. The drug-sensitive representatives that are the subject of this report then rapidly diversified in regional MDR and XDR sublineages (21, 22, 30, 38–43, 45–47).

## MATERIALS AND METHODS

### Strains and clinical data.

The *P. aeruginosa* strain collection consists of isolates from the inanimate environment, mainly from freshwater habitats, acute animal and human infections, and chronic airway infections of people with CF, bronchiectasis, or COPD (17). P. aeruginosa strains RN3 and 60P57PA had been isolated from the airways of patients with CF (28) and COPD (29), respectively. The origins of the panels of the 13 clone D421 (ST253) and clone F46D (ST235) strains utilized for this study are listed in Data Set S1 in the supplemental material. Secondary subcultures were stored in triplicates as glycerol stock cultures at −80°C. The strains were genotyped with a low-resolution microarray that represents the conserved core genome with 16 informative SNPs and the variable accessory genome with 42 marker genes (23). Data Set S1 lists the 2,882 entries of genotyped strains.

Information on patient characteristics (underlying disease and age at infection with P. aeruginosa) and on antipseudomonal chemotherapy was extracted from the patients’ records. The study has been approved by the ethics committee of Hanover Medical School (study number 3739). Written informed consent was obtained from patients and their parents, if applicable.

### Phenotypic bacterial assays.

**(i) Colony morphology.** Colony morphology was visualized on blood agar or Congo red (40 μg/ml) agar plates. Two microliters of P. aeruginosa cultures from late stationary phase was inoculated onto the plate and incubated at 37°C for 36 h.

**(ii) Hemolysis and autolysis.** Hemolytic bacterial activity was tested by dropping 2 μl of an overnight culture onto a blood agar plate. After 48 h of incubation at 37°C, hemolysis was evident by a greenish or yellowish circle. Autolysis was judged from the same plate after 24, 48, and 168 h by the appearance of small holes of lysed cells within the bacterial lawn.

**(iii) Plate assays of siderophore and protease secretion.** Siderophore secretion was assessed by an orange halo around colonies grown for 24 h at 37°C on chrome azurol S plates (58). Secretion of casein-degrading proteases was examined by growing the analyzed P. aeruginosa strains on M9 agar plates supplemented with 0.8% (wt/vol) casein (59).

**(iv) Motility assays.** To assess bacterial swimming, 2 μl of bacterial suspension was stab inoculated into 0.3% LB agar plates, the plate was incubated for 12 h at 37°C, and the circular turbid zone of cell migration was measured. To assess swarming, 2 μl of a bacterial overnight culture was dropped onto a 0.5% LB agar plate. The swarming zone was measured after 12 h of incubation at 37°C (60). Twitching motility was assayed according to the protocol by Alm and Mattick (61). Bacteria from liquid culture were applied to the subsurface of 1% LB agar allowing bacterial locomotion at the agar/plastic interface at 37°C. After 12 h, the twitching zone on the petri dish was quantified by Coomassie staining (0.05% Coomassie brilliant blue R250 solution, 50% methanol, 10% acetic acid, 40% H_2_O).

### Fermentation.

A loop of stored bacteria was streaked onto a tryptone-soybean agar plate and incubated for 16 h at 37°C. Loops of bacteria were inoculated into two flasks of 30 ml tryptone soybean broth, each adjusted to an optical density at 600 nm (OD_600_) of 0.25. For preculturing, the bacterial suspension was incubated for 1 h at 37°C with shaking (125 rpm). Thereafter, an aliquot of the bacterial suspension equivalent to 10^7^ CFU/ml was added to 1.5 liters tryptone soy broth in a BIOSTAT Bplus 2-liter CC fermenter (Sartorius BBI Systems, Melsungen, Germany). Bacteria were cultured at 37°C and pH 7.0 under constant agitation (400 rpm). Compressed air of 0.3 liters/min was continuously injected with a microsparger (pore size, 10 μm). The pH was held constant by addition of either 5 M H_3_PO_4_ or 1 M NaOH. Oxygen saturation of broth calibrated to compressed air was monitored by polarography (electrode set 12/215-L-HM-UniVessel; Hamilton, Bonaduz, Switzerland). Redox potential of the culture was recorded with a redox electrode (Pt4805-DPAS-SC-K8S/200; Mettler Toledo, Columbus, OH). Data were stored every 6 min. Every 60 min, 1 ml was removed from the culture for the determination of CFU and OD_600_. When oxygen saturation had dropped for the first time to 50%, 3 aliquots of 5 ml each were removed for RNA extraction as a surrogate of gene expression of mid-exponential phase. The second set of samples was harvested 2 h later during the early stationary phase (see Fig. 5). Fermentations were executed in duplicates.

### RNA extraction.

Eight milliliters of RNAprotect (Qiagen, Hilden, Germany) was added to each RNA extraction aliquot and centrifuged for 10 min at 5,000 × *g*. The pellet was frozen at −80°C until use. For RNA isolation, the bacterial pellet was resuspended in 400 μl Tris-EDTA (TE) buffer containing 1.5 mg/ml lysozyme, and cells were lysed for 15 min at room temperature. Thereafter, RNA was prepared with the RNeasy minikit (Qiagen, Hilden, Germany) according to the protocol of the manufacturer. The RNA preparation was purified from contaminating DNA by two cycles of on-column DNase I digestion. RNA was eluted from the column with 2 × 75 μl H_2_O. After addition of 2 μl RNasin, the solution was stored at −80°C. The quality of the RNA preparation was checked with the Agilent 2100 Bioanalyzer (Agilent Technologies, Santa Clara, CA).

### RNA sequencing and data evaluation.

The fraction of ribosomal RNAs was depleted from the RNA preparation with the Ribo-Zero magnetic kit (Illumina, San Diego, CA). Strand-specific cDNA libraries were prepared by GATC Biotech(Constance, Germany) and sequenced on an Illumina HiSeq 2000 instrument.

Strand-specific reads were mapped to the PA14 and NCGM2.S1 reference using bwa (62). Differential expression was analyzed using the DESeq2 (63) and EDAseq (64) R packages. Filtering of candidates was based on the fold change and adjusted *P* value.

### DNA sequencing, read processing, and genomic mapping.

Artificial reads with a length of 75 bp were generated from the reference sequence NCGM2.S1 using Picard Toolkit (http://broadinstitute.github.io/picard/). The strains 60P57PA and RN3 were sequenced as tagged paired-end libraries on an Illumina Genome Analyzer II by GATC Biotech (Constance, Germany) (13). The genomes of all other F46D strains were sequenced on the Illumina NextSeq 500/550 platform (high-output kit v2.5, 75 cycles, single-end reads, number 20024906). The flow cell was underclustered (1.3 pM instead of default 1.5 pM) to prevent cluster overlaps. Sequencing libraries were prepared with an NEBNext Ultra II DNA library prep kit for Illumina (E7645 and E7103), with NEBNext unique dual index primer pairs and 12 PCR cycles. Clone D421 strains had been sequenced previously on a SOLiD 5500 XL instrument within the frame of another project (35).

After sequencing, duplicated reads were removed using FastUniq (65), and adaptive read trimming was performed with Trimmomatic (66). SNP comparison was performed based on read alignment to the PA14 or NCGM2.S1 reference genomes with the bwa mem algorithm (62). SNPs were called with the function callvariants of BBMAP (https://sourceforge.net/projects/bbmap/), and SNP classification was performed using SnpEff 4.3t (67). Visual comparison of candidate loci was conducted with IGV (68). Accessory genome comparison was conducted using *de novo* assembled sequence contigs and scaffolds generated by the software Newbler (version 2.8) (69). Heat maps were generated with the R package pheatmap (70).

### Data availability.

The genome and transcriptome sequencing data sets have been deposited in the European Nucleotide Archive (ENA) hosted by the European Bioinformatics Institute EMBL-EBI (www.ebi.ac.uk). The study accession numbers of the ST235 and ST253 read data sets are PRJEB40495 and PRJEB40497. The RNA-seq data were assigned to the study “Transcriptome comparison of the most common Pseudomonas aeruginosa clones D421 and F46D” (study accession no. PRJEB37218).
